# Delayed Metastatic Cholangiocarcinoma to the Stomach With Imaging Characteristics Resembling Gastrointestinal Stromal Tumor: A Case Report and Review of the Literature

**DOI:** 10.7759/cureus.41785

**Published:** 2023-07-12

**Authors:** Abtin Jafroodifar, Janet Tam, Zohaib V Khan, Michele Lisi

**Affiliations:** 1 Radiology, State University of New York Upstate Medical University, Syracuse, USA; 2 Radiology, Touro College of Osteopathic Medicine, New York, USA

**Keywords:** gastrointestinal stromal tumor, cholangiocarcinoma, magnetic resonance imaging, metastatic cholangiocarcinoma, intrahepatic tumor, ct (computed tomography) imaging, gastrointestinal stromal tumor (gist)

## Abstract

Intrahepatic cholangiocarcinoma (ICC) is a relatively rare subtype of cholangiocarcinoma, and there has been an increasing incidence of ICC in Western countries in recent years. Surgical resection is the most effective treatment for ICC. However, overall outcomes are extremely poor given that most patients are diagnosed at an advanced stage, and postoperative ICC recurrence is still very high despite hepatic resection. We report a case of metastatic ICC to the stomach presenting after resection of the original tumor, with imaging characteristics highly resembling gastrointestinal stromal tumor (GIST) on imaging. Reported cases of metastatic ICC to the liver are sparse. Given that there is a significant difference in the survival rate between metastatic cholangiocarcinoma and other tumors arising from the gastrointestinal tract, including GISTs, it is important to delineate the differences via imaging features. We further discuss the imaging characteristics of intrahepatic ICC, comparing and contrasting it to other gastric tumors.

## Introduction

Metastasis of intrahepatic cholangiocarcinoma (ICC) is commonly found in the lungs, brain, skin, bones, and other organs but very rarely found in the stomach. There is a paucity of cases demonstrating gastric metastasis of ICC, with only five previously reported case reports found on PubMed. In four of the cases, gastric metastasis was found to exist concurrently with cholangiocarcinoma while evaluating for vague abdominal symptoms like epigastric pain and dysphagia [1,2]. Imamura et al.'s case report is the only one that discusses recurrent cholangiocarcinoma found in the stomach post-surgical resection, reflecting distant metastasis [3]. This case represents the sixth overall reported case of ICC distant metastatic disease to the stomach, and the second to demonstrate gastric metastatic disease after surgical resection of the original ICC tumor.

We report a rare case of metastatic ICC to the stomach that was discovered three years after initial surgical management, including cholecystectomy and left hepatectomy with caudate lobe resection for the primary tumor. On the surveillance CT imaging, the exophytic gastric mass was initially thought to resemble a gastrointestinal stromal tumor (GIST) given its both endo- and exophytic rounded configuration.

This report and literature review highlight the imaging characteristics of ICC as well as other gastric tumors. By describing the radiologic findings in our case, we hope to increase awareness of this rare occurrence and explain the importance of it being on a radiologist’s differential diagnosis.

## Case presentation

**Figure 1:**
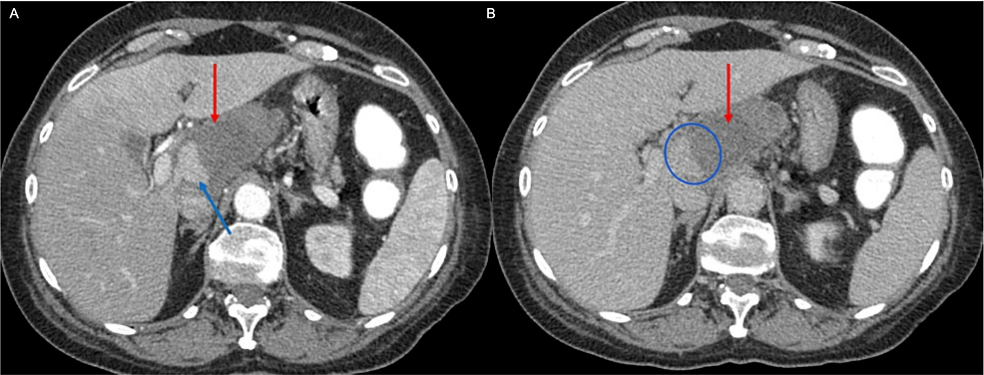
Contrast-enhanced axial images of the abdomen during the arterial (A) and portovenous (B) phases demonstrate a hypodense mass involving the caudate lobe of the liver (segment 1) (red arrows in A and B). The mass demonstrates internal vascularity and hemorrhagic components (blue arrow in A) with increased pooling (blue circle in B).

A 63-year-old Caucasian woman presented with progressive pain in the upper abdomen for a few weeks after sustaining trauma to the abdomen. The initial contrast-enhanced CT scan of the abdomen and pelvis demonstrated a 7-cm hemorrhagic lesion in the left hepatic lobe, which was originally thought to be a ruptured hepatic adenoma (Figure 1).

**Figure 2:**
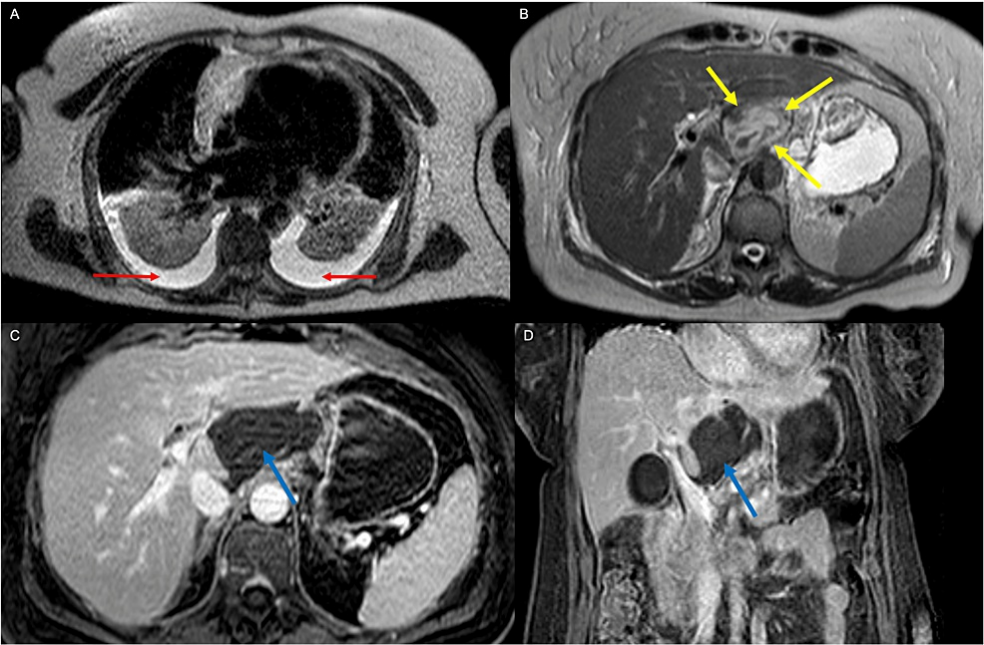
Axial MRI of the abdomen. T2-weighted images (A, B) demonstrate bilateral pleural effusions (red arrows in A) as well as a mixed-density heterogeneous mass arising from the caudate lobe of the liver (yellow arrows in B). Axial (C) and coronal (D) contrast-enhanced images demonstrated curvilinear enhancement of the caudate lobe mass with internal signal suggestive of proteinaceous or hemorrhagic components (blue arrows in C and D).

MRI of the abdomen with and without contrast two days later revealed a 5.0 x 3.7 cm heterogeneous mass with some curvilinear enhancement in the caudate lobe of the liver. There were also moderate bilateral pleural effusions with overlying atelectasis and/or airspace disease (Figure 2). A tissue biopsy of the mass was suggested for further evaluation but was deferred for en mass resection. The patient was discharged home with a plan for elective operative resection of the mass.

During the surgery for a caudate lobe resection and cholecystectomy, the surgery was expanded to include a left hepatectomy due to the fact that her tumor was shown to be more extensive than originally believed. Pathology results were consistent with ICC. The patient subsequently completed adjuvant chemotherapy. Interval scans every six months were recommended for surveillance, which the patient adhered to up to two years from her original treatment. She was lost to follow-up for one year, but eventually returned to the clinic at which point her surveillance was re-initiated.

**Figure 3:**
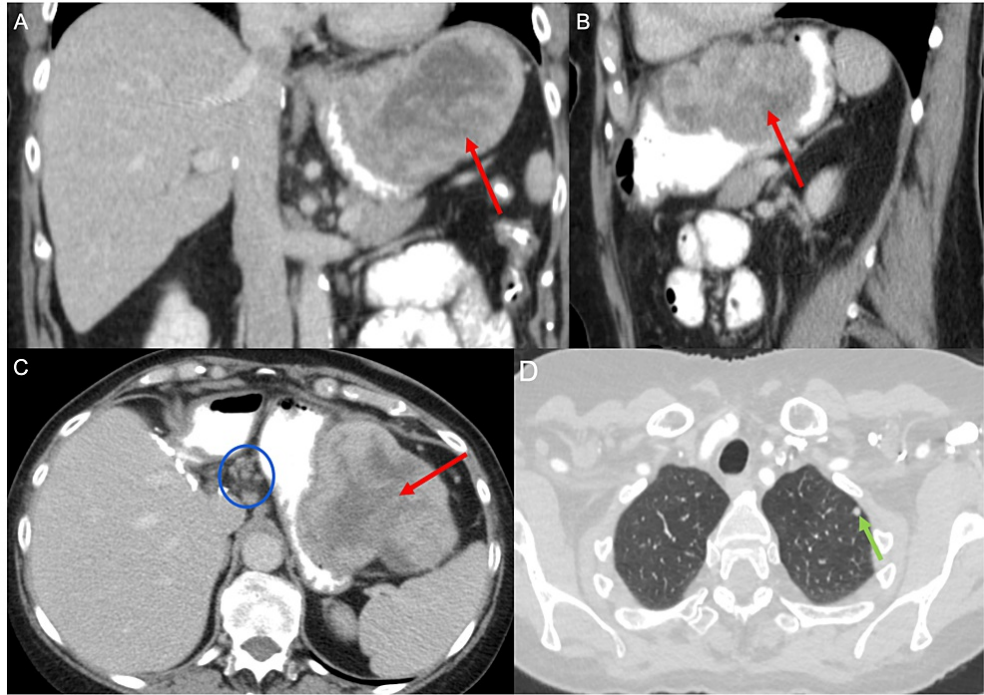
Oral and intravenous contrast-enhanced CT images of the abdomen in coronal (A), sagittal (B), and axial (C) views demonstrate a heterogeneous gastric mass that is both effacing the stomach lumen as well as exophytically extending beyond the gastric wall (red arrows in A, B, and C). Scattered lymph nodes are seen at the celiac axis (blue circle in C). Axial CT image of the thorax (D) demonstrates a new left upper lobe lung nodule (green arrow in D) measuring 9 mm.

Contrast-enhanced CT of the abdomen and pelvis performed at her re-initiation of surveillance demonstrated a 9.8 cm exophytic gastric mass along the greater curvature of the stomach, along with lymphadenopathy at the celiac station that was not present on imaging one year prior (Figure 3). The patient also now reported symptoms of early satiety, food aversion, and worsening heartburn.

**Figure 4:**
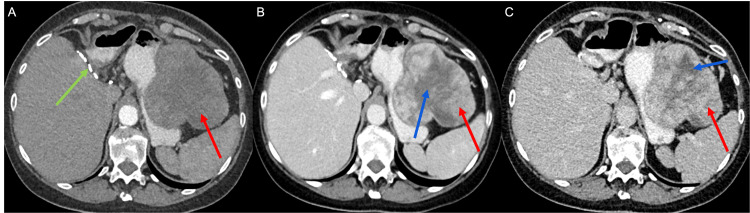
Axial CT images of the abdomen without contrast (A) during the arterial phase (B) and during the portovenous phase (C) demonstrate an exophytic heterogeneous mass involving the greater curvature of the stomach (red arrows in A, B, and C). There is effacement of the gastric lumen, similar to the imaging a month prior. The mass enhances on the arterial phase and has internal streaky hypodensities on the arterial (blue arrow in B) and portovenous (blue arrow in C) phases. No calcifications are present as noted by the non-contrast image; surgical staples from prior left hepatectomy are present (green arrow in A).

The patient insisted on repeat CT imaging one month later before starting therapy. This examination showed a well-circumscribed lesion with heterogeneous enhancement on the arterial and venous phase images and central necrosis, with the appearance suggesting a gastrointestinal tumor (Figure 4). Mass effect was also seen on the gastric lumen at the fundus and proximal body. Scattered subcentimeter lymph nodes were identified within the porta hepatis and along the lesser curvature of the stomach. CT of the thorax on the same day revealed new nodules in the upper lobe of the left lung, with the largest measuring 9.0 mm in maximum dimension, which was concerning for a metastatic lesion (Figure 3D).

Esophagogastroduodenoscopy (EGD), endoscopic ultrasound (EUS), and fine needle aspiration (FNA) were performed. On EGD, an approximately 10 mm mildly irregular and slightly raised mucosal patch was found on the duodenal sweep, and biopsies were taken. On EUS, three areas of concern for malignancy were identified. There was a large 105 mm x 60 mm heterogeneously mildly hyperechoic heterogeneous abdominal mass impressing upon the stomach from the outside and the gastric wall with a satellite lesion measuring 13.2 mm x 9.5 mm immediately adjacent to and contiguous with this mass. Within this lesion, there were areas of suspected necrosis. The lesion was inseparable from the gastric wall at EUS evaluation. There was a 21.8 mm x 19.9 mm hypoechoic malignant-appearing perihepatic mass adjacent to the proximal inferior vena cava. There was also an 18.1 mm x 12.6 mm mildly hyperechoic spherical mass lesion with a 3.7 mm pedicle that appeared to penetrate through the wall of the most cephalad portion of the inferior vena cava adjacent to the tricuspid valve. FNA of the large abdominal mass and perihepatic mass was performed, but FNA was not performed for the mass adjacent to the tricuspid valve, as it was a high-risk site and likely would not change the management of care. Pathology results indicate that both the large gastric and perihepatic masses obtained during EUS were positive for carcinoma cells and tissue fragments comparable with pancreaticobiliary ductal adenocarcinoma, reflecting metastatic cholangiocarcinoma.

The patient felt grateful for the three years of life she has had since her initial diagnosis. Having witnessed her husband suffer from the effects of chemotherapy for several years, she ultimately decided to pursue hospice instead of further treatment for her metastatic disease.

## Discussion

There is a paucity of cases demonstrating gastric metastasis of ICC, with only five previously reported case reports found on PubMed. In four of the cases, gastric metastasis was found to exist concurrently with cholangiocarcinoma while evaluating for vague abdominal symptoms like epigastric pain and dysphagia [1,2]. Imamura et al.'s case report is the only one that discusses recurrent cholangiocarcinoma found in the stomach post-surgical resection, reflecting distant metastasis [3]. In our case, we report another case of postoperative metastatic cholangiocarcinoma to the stomach.

ICC is a rare subtype of cholangiocarcinomas and makes up less than 10% of cholangiocarcinoma cases [4]. ICC arises from bile duct epithelial cells above the secondary bile duct. It has a very poor prognosis due to the lack of symptoms during the early onset of the disease leading to the fact that patients are usually diagnosed at a very advanced stage. Even after hepatic resection, the overall outcome is poor, with a five-year survival rate of 30-35% [5,6]. More than 60% of people may develop ICC recurrence after hepatic resection, with the most common locations of metastasis being the lungs (24%), peritoneum (18%), and bones (14%) [1,7]. Other locations include the brain, breast, skin, colon, and blood system [1,8]. Classically, ICC may spread via direct invasion, the lymphatic system, hematogenously, or a combination of these [4]. Due to the location of the metastasis, we believe that our case most likely is a result of direct invasion into the visceral peritoneum of the stomach. When there is distant metastasis, the survival rate is significantly lower than intrahepatic recurrence or locoregional recurrence. In their study, Chan et al. reported that the one-year survival rate in intrahepatic recurrence, locoregional recurrence, and distant metastasis was 64.3%, 41.3%, and 16.9%, respectively [6].

ICC most often presents as a malignant mass lesion in a noncirrhotic liver. Specifically, contrast-enhanced cross-sectional imaging is helpful for differentiating ICC from hepatocellular carcinoma. Cholangiocarcinoma will have progressive contrast uptake throughout both arterial and venous phases because it is sclerosing, composed of fibrous tissue, and receives blood supply from the portal vein [1,4,9]. While the peripheral portion of ICC contains many viable tumor cells, the central portion has coagulative necrosis with few viable tumor cells and fibrous stroma. On CT with contrast, ICC classically appears as a hypoattenuating mass with irregular peripheral enhancement in the arterial phase and slow central enhancement shown on delayed images. On MRI, ICC typically appears hypointense on T1-weighted imaging and hyperintense on T2-weighted imaging, with contrast enhancement patterns like those found on CT [10]. Both CT and MRI are the imaging modalities of choice for diagnosing cholangiocarcinoma, but both combined have a higher sensitivity and specificity than each of them alone [11]. It is believed that the more sclerosing tumors intend to invade the ductal wall earlier, thus leading to lower cure rates. Our patient most likely had the sclerosing-type ICC, resulting in sudden and aggressive metastasis that became obviously apparent in 12-month follow-up imaging. Although there are no clear guidelines regarding surveillance imaging of ICC following surgery, we believe that more frequent monitoring at least every six months is necessary. In the future, it may be beneficial to consider using 18F-fluorodeoxyglucose positron emission tomography (18F-FDG PET)/CT, which is a less-conventional but promising diagnostic tool for assessing recurrent ICC due to its ability to detect metabolic and post-therapeutic changes.

In our patient, it was difficult to distinguish gastric metastasis of cholangiocarcinoma from primary gastric cancer based on clinical and radiologic features. The large metastatic lesion found impressing on the gastric wall was initially thought to resemble a GIST due to its imaging characteristics. GISTs are often very large with areas of necrosis and cystic degeneration. On CT, these tumors have varying densities and show patchy enhancement after contrast is administered [12,13]. GISTs are thought to be locally invasive, and thus frequently can reach large sizes before causing any pain or signs of bowel obstruction. They are usually found on imaging incidentally. In our patient, surveillance CT imaging reflected this pattern of imaging, and clinical symptoms could be explained by the tumor’s mass effect. Due to the nonspecific and overlapping imaging features found in both GISTs and ICCs, it is important to consider the clinical history and further evaluation by endoscopy and endoscopic ultrasound. This allows a more direct look at the characteristics of the mass surrounding the gastric wall and a route to take image-guided biopsy samples of the mass for analysis of the histopathologic features.

In addition to GISTs, there are mesenchymal tumors of the stomach that may appear very similar to ICC on imaging. For example, non-GIST sarcomas such as liposarcomas and leiomyosarcomas are large, aggressive tumors with heterogeneous enhancement and areas of necrosis [14]. Histopathologic evaluation is very important for the evaluation of these gastric tumors because there are great differences in treatment response to chemotherapy. GISTs are normally positive for c-KIT, CD117, CD34, and DOG1 tumor markers, while non-GIST sarcomas are generally negative. GISTs will respond well to imatinib, a tyrosine kinase inhibitor, whereas non-GIST sarcomas will not [14,15]. Another neoplasm that has very similar radiologic findings is the inflammatory myofibroblastic tumor (IMFT), which frequently can be found in the abdominal cavity with invasion through the abdominal wall. On CT, IMFTs are seen as large, aggressive intramural masses that enhance with contrast and have a heterogeneous pattern [14].

Other gastric neoplasms that are worth contrasting include gastric adenocarcinoma, gastric lymphoma, and gastric carcinoid tumors. Gastric adenocarcinoma is the most common gastric malignancy. On CT, adenocarcinomas have focal wall thickening with or without ulceration, polypoidal mass, or diffuse infiltration (termed linitis plastica) [16]. Gastric lymphomas only represent 1-5% of malignant stomach tumors and are predominantly B-cell non-Hodgkin's lymphomas. They classically have a segmental or diffuse gastric wall thickening of more than 1 cm. The diffuse homogeneous wall thickening, less pronounced enhancement, and preservation of perigastric fat planes can help distinguish gastric lymphoma from gastric adenocarcinoma [16,17]. Lastly, type III gastric carcinoid tumors, which are the least common type of gastric carcinoids, can appear similar to ICC on imaging. CT features for gastric carcinoids include large solitary hypervascular masses with exophytic features. In MRI, T1-weighted images may show hypointensity and T2-weighted images may demonstrate the mass to be slightly hyperintense [16].

Cross-sectional imaging of the chest and abdomen should be carefully assessed by a radiologist to guide treatment in patients with ICC. The extent of the tumor burden, presence of metastatic lesions, lymph node involvement, and vascular/lymphatic involvement should be evaluated to determine whether the patient is a surgical candidate. It is important to note that the probability of curative surgical resection is low and the median survival time by intention-to-treat analysis of surgical resectable lesions on imaging is 36 months [4]. This is partly due to the high incidence of tumor recurrence for this disease. Although the effectiveness of locoregional therapy has not been studied in high-quality research studies, these options may be explored as a palliative approach for those who are not surgical candidates. Treatments such as transarterial chemoembolization (TACE) and Y-90 are reasonable treatment options with demonstrated tolerability and potential benefit [4]. Kuhlmann et al. reported that TACE with drug-eluting beads, specifically irinotecan-eluting beads, may have similar effectiveness as systemic chemotherapy [18]. This emphasizes the importance of the radiologist’s role in accurately reporting imaging findings for these patients as well as their role in clinical management via performing palliative treatments.

## Conclusions

ICC is a devastating disease with a very poor prognosis and poorly understood metastatic behavior. ICCs tend to recur despite apparent complete surgical resection and treatment, lending to their overall morbidity and mortality. Our case presents an instance of recurrence of ICC to the stomach, with the metastatic focus sharing very similar findings to a GIST. Additionally, other gastric tumors share similar imaging findings to ICCs and GISTs as well, including adenocarcinoma, lymphoma, and carcinoid tumors.

It is important to appropriately recognize metastatic ICC and raise the possibility by the interpreting radiologist, including describing the extent of the tumor burden, presence of metastatic lesions, lymph node involvement, and vascular/lymphatic involvement to properly guide clinical management and help the primary providers determine whether the patient is a surgical candidate. Additionally, potential treatment modalities include endovascular therapy, such as chemoembolization. Early recognition of ICC can lead to hastening of therapy to allow more time to patients.
